# i2APP: A Two-Step Machine Learning Framework For Antiparasitic Peptides Identification

**DOI:** 10.3389/fgene.2022.884589

**Published:** 2022-04-27

**Authors:** Minchao Jiang, Renfeng Zhang, Yixiao Xia, Gangyong Jia, Yuyu Yin, Pu Wang, Jian Wu, Ruiquan Ge

**Affiliations:** ^1^ School of Computer Science and Technology, Hangzhou Dianzi University, Hangzhou, China; ^2^ Shandong Provincial Hospital Affiliated to Shandong First Medical University, Jinan, China; ^3^ Computer School, Hubei University of Arts and Science, Xiangyang, China; ^4^ MyGenostics Inc., Beijing, China

**Keywords:** antiparasitic peptides, feature representation, maximum information coefficient, feature selection, T-distributed stochastic neighbor embedding

## Abstract

Parasites can cause enormous damage to their hosts. Studies have shown that antiparasitic peptides can inhibit the growth and development of parasites and even kill them. Because traditional biological methods to determine the activity of antiparasitic peptides are time-consuming and costly, a method for large-scale prediction of antiparasitic peptides is urgently needed. We propose a computational approach called i2APP that can efficiently identify APPs using a two-step machine learning (ML) framework. First, in order to solve the imbalance of positive and negative samples in the training set, a random under sampling method is used to generate a balanced training data set. Then, the physical and chemical features and terminus-based features are extracted, and the first classification is performed by Light Gradient Boosting Machine (LGBM) and Support Vector Machine (SVM) to obtain 264-dimensional higher level features. These features are selected by Maximal Information Coefficient (MIC) and the features with the big MIC values are retained. Finally, the SVM algorithm is used for the second classification in the optimized feature space. Thus the prediction model i2APP is fully constructed. On independent datasets, the accuracy and AUC of i2APP are 0.913 and 0.935, respectively, which are better than the state-of-arts methods. The key idea of the proposed method is that multi-level features are extracted from peptide sequences and the higher-level features can distinguish well the APPs and non-APPs.

## Introduction

Parasites are a very common source of disease. Parasitic diseases can affect almost all living things, including plants and mammals. The effects of parasitic diseases can range from mild discomfort to death (Momčilović et al., 2019). It is estimated that one billion people worldwide are infected with ascariasis, although it is usually harmless. Necator americanus and Ancylostoma duodenale can cause hookworm infections in humans, resulting in anemia, malnutrition, shortness of breath and weakness. This infection affects about 740 million people in the developing countries, including children and adults (Diemert et al., 2018). Malaria is very harmful to humans. It causes 300 to 500 million illnesses and about 2 million deaths each year, with about half of those deaths occurring in children under the age of 5 (Barber et al., 2017). The main method of treating parasitic diseases today is the use of antibiotics (Zahedifard and Rafati, 2018). However, frequent use of antibiotics can increase parasite resistance and even have some undetected side effects (Ertabaklar et al., 2020). Studies have found that anti-parasite peptide (APP) can effectively inhibit the growth of parasites and even kill them (Lacerda et al., 2016). Anti-parasite peptides are usually composed of 5–50 amino acids and are relatively short in length. They are usually changed by antimicrobial peptides (AMPs) (Mehta et al., 2014). APPs can kill parasites by destroying the cell membrane of the parasite or inhibiting the reductase in the parasite (Bell, 2011; Torrent et al., 2012). Therefore, it is very important to be able to identify APPs.

In the past few years, many methods for predicting functional peptides based on machine learning have been proposed, such as AAPred-CNN (Lin et al., 2022) for anti-angiogenic peptides, mAHTPred (Manavalan et al., 2019) for anti-hypertensive peptides, AVPIden (Pang et al., 2021) for anti-viral peptides. PredictFP2 can predict fusion peptide domains in all retroviruses (Wu et al., 2019). AMPfun (Chung et al., 2020) and PredAPP (Zhang et al., 2021) are proposed for antiparasitic peptides identifiction. Based on random forests, the AMPfun tool can be used to identify anticancer peptides, APP, and antiviral peptides. AMPfun can be used to characterize and identify antimicrobial peptides with different functional activities, but the prediction results for APPs are not very good. In 2021, (Zhang et al., 2021) proposed PredAPP, a model for predicting antiparasitic peptides using an under sampling and ensemble approach. A variety of data under sampling methods are proposed for data balance. This model adopts an ensemble approach, combining 9 feature groups and 6 machine learning algorithms, and finally achieves good results, but there is still room for improvement.

In this work, we propose a new model named i2APP for identifying APPs, which uses a two-stage machine learning framework. In the first stage, we extract dozens of feature groups for each peptide sequence, and then build the first-layer classifiers with these feature groups. The outputs of the first-layer classifiers are used as the higher-level features. What’s more, MIC (Kinney and Atwal, 2014; Ge et al., 2016) is used here to filter out the insignificant features. In the second stage, with the higher-level features, we build the second-layer classifier, whose outputs are the final results of identifying APPs. Through independent test, we will find that the proposed model is better than the state-of-arts methods in most metrics. The tool i2APP is available at https://github.com/greyspring/i2APP.

## Materials and Methods

### Datasets

A benchmark dataset is the premise for an effective and reliable model. To train our model and compare it with others, the dataset studied by (Zhang et al., 2021) were used in this work, in which 301 APPs were used as positive samples and 1909 non-APPs were negative ones. For the positive samples, 301 APPs were taken out as positive training samples, and the remaining 46 APPs were used as positive testing samples. 46 non-APPs were randomly selected from the negative samples as negative testing samples, and the remaining 1863 non-APPs were used as negative training samples. In this way, 255 APPs and 1863 non-APPs constituted the original training set, and 46 APPs and 46 non-APPs constituted the testing set. Since the samples in the training set are very unbalanced, we use random under sampling (Tahir et al., 2012; Stilianoudakis et al., 2021) on the training set and get 255 APPs and 255 non-APPs to constitute the final training set. For the sake of simplicity, the final training dataset is marked as T255p + 255n, and the testing dataset is marked as V46p + 46n.

**FIGURE 1:**
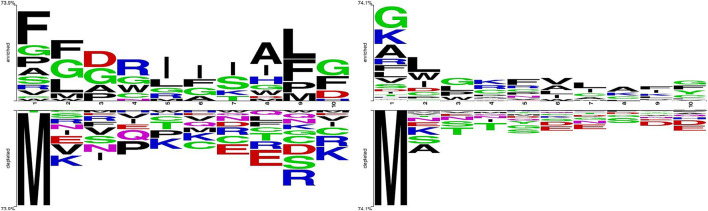
Different distribution between APP and non-APP sequences. **(A)** V46p+46n **(B)** T255p+1863n.

We take out the 5 amino acids at the N-terminus and C-terminus of each peptide sequence to compare the differences between positive and negative samples by Two Sample Logo application (Schneider and Stephens, 1990; Crooks et al., 2004), which calculates and visualizes the differences between two sets of aligned samples of amino acids or nucleotides. At each position in the aligned groups of sequences, statistically significant amino acid symbols are plotted using the size of the symbol that is proportional to the difference between the two samples. It can be seen from the comparison in Figure 1 that the amino acid composition at both ends of the APPs and non-APPs sequences have some differences, so it can be considered to extract features from both ends of peptide sequence to distinguish the two types of samples.

### Features Representation

Good features are beneficial to the training of machine learning models and obtain good prediction performance. The classification of peptides mainly depends on the feature set constructed by the structural and functional properties. Extracting features from peptide sequences that effectively reflect their sequence pattern information is a challenging problem. In this study, we extract 18 kinds of physicochemical features from the peptide sequences, some of which contain very important information, such as functional domains, gene ontology and sequential evolution, etc (Liu et al., 2015; Liu et al., 2017). Thus 18 groups of sequence-based features will be obtained for each peptide sequence.

In addition, the N-terminus and C-terminus of a protein or peptide often have very important biological function, so we also extract features from the both ends of peptide sequence. In this study, we take out a fragment with three or five amino acids at the N-terminus or C-terminus of a peptide sequence, and use 12 types of feature extraction method for this fragment (Jing et al., 2019). In such a way, 48 groups of terminus-based features will be obtained for each peptide sequence.

All these feature extraction methods are listed in Table 1.

**TABLE 1:** Peptide sequence features.

	Features
Sequence-based	Basic Kmer (kmer)
	Distance-based Residue (DR)
	Distance Pair (DP)
	Auto covariance (feature-AC)
	Auto-cross covariance (ACC)
	Cross covariance (feature-CC)
	Physicochemical distance transformation (PDT)
	Parallel correlation pseudo amino acid composition (PC-PseAAC)
	Series correlation pseudo amino acid composition (SC-PseAAC)
	General parallel correlation pseudo amino acid composition (PC-PseAAC-General)
	General series correlation pseudo amino acid composition (SC-PseAAC-General)
	Select and combine the nmost frequenct aminoacids according to their frequencies (Top-n-gram)
	Profile-based Physicochemical distance transformation (PDT-Profile)
	Distance-based Top-n-gram (DT)
	Profile-based Auto covariance (AC-PSSM)
	Profile-based Cross covariance (CC-PSSM)
	Profile-based Distance-based Top-n-gram (PSSM-DT)
	Profile-based Auto-cross covariance (ACC-PSSM)
Terminus-based	One_hot
	One_hot_6_bit
	Binary_5_bit
	Hydrophobicity_matrix
	Meiler_parameters
	Acthely_factors
	PAM250
	BLOSUM62
	Miyazawa_energies
	Micheletti_potentials
	AESNN3
	ANN4D

### Computational Models

**FIGURE 2:**
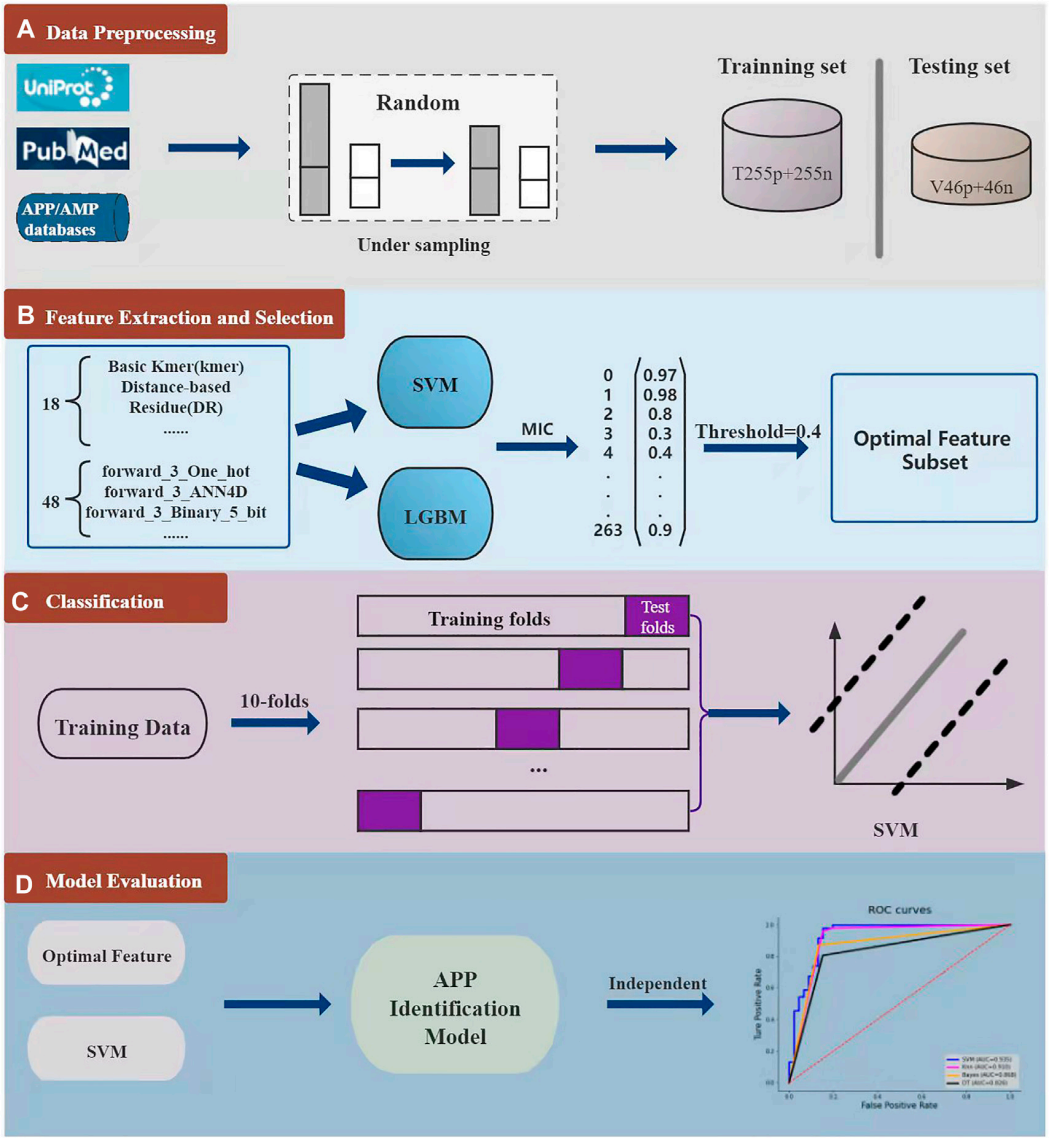
The whole model consists of four parts. The first part is the collection, division and down sampling of the dataset. The second part is feature extraction and feature selection for each peptide sequence. The third part is to analyze the effect of different classifiers through 10-fold cross-validation. In the fourth part, the proposed model is evaluated through independent test.

As shown in Figure 2, the overall framework of i2APP includes four main steps. As a first step, the benchmark datasets are collected from various databases and literates, and then divided into training dataset and testing dataset. To get a balanced training dataset, the random under sampling procedure is performed on the negative training samples. In the second step, we adopt 18 types of feature extraction methods on the whole peptide sequence to get 18 groups of sequence-based features, and 12 types of feature extraction methods on the N-terminus and C-terminus of peptide sequence. Considering that all peptide sequences are at least 5 residues in length, we take 3 and 5 residues at both ends of the sequence. So, a total of 48 groups of terminal-based features are extracted. For each feature group, SVM and LGBM are trained respectively, and 132 probability outputs are got for each peptide sequence. These probabilities can be seemed as higher-level features for further classification. What’s more, the probability greater than 0.5 is recorded as 1, and the probability less than 0.5 is recorded as 0. These binarized values help remove noise from the model. Stacking the probabilities and their binarized values, a total of 264 higher-level features are obtained. However, these higher-level features may have information redundancy, so a feature selection method is needed here to filter out the superfluous ones. In this study, the maximum information coefficient (MIC) is calculated for each feature, and the threshold is set to 0.4, that is, only the feature with the MIC value greater than 0.4 is retained. The third step is to use ten-fold cross-validation to select the best classifier based on the reduced higher-level feature set. The candidate include the popular classifiers, such as SVM, Bayes (Jahromi and Taheri, 2017), Decision Tree (DT) (Wang et al., 2019), K-Nearest Neighbor (KNN) (Wang et al., 2017), Random Forest (RF), Adaboost (Ada) and so on. In the fourth step, we test the effect of the proposed model on an independent test dataset, and compare its performance with other models. In this work, we used the scikit-learn package (Pedregosa et al., 2011) to implement all classifiers.

### Evaluation

In order to evaluate the results of the final classification and facilitate comparison with other models, we used five commonly used indicators in bioinformatics research (Luo et al., 2019; Yang et al., 2021), including specificity (SP), sensitivity (SN), F1 score (F1), Matthew correlation coefficient (MCC) and accuracy (ACC). The specific calculation formula of these measured values is as follows:

$$Sp=\frac{TN}{TN+FP}$$

$$Sn=\frac{TP}{TP+FN}$$

$$F1=\frac{2TP}{2TP+FP+FN}$$

$$Acc=\frac{TP+TN}{TP+TN+FP+FN}$$

$$MCC=\frac{TP\cdot TN-FP\cdot FN}{\sqrt{\left(TP+FP\right)\cdot \left(TP+FN\right)\cdot \left(TN+FP\right)\cdot \left(TN+FN\right)}}$$

Where TP means the number of APPs correctly predicted by the model; TN means the number of non-APPs that the model correctly predicts; FP means the number of non-APPs that the model mispredicts; FN means the number of APPs that the model mispredicts. In addition, we also use other metrics to evaluate the performance of i2APP, including receiver operating characteristic (ROC) curve (Fawcett, 2006), the area under the ROC curve (AUC) (Lobo et al., 2008), precision-recall (PR) curve (Davis and Goadrich, 2006), and the area under the PR curve (AUPR).

## Results

### Effects of Different Classifiers

First, we fix the classifier of the second layer as SVM because it is very effective in small sample learning, and then compare the different classification models in the first layer. Through cross-validation experiments, it is found that the effects of SVM and LGBM are better, so we use these two classification models in the first layer. Now we can compare different classifiers in the second layer. As can be seen from Table 2, different classifiers are tested on the training dataset T255p + 255n through ten-fold cross-validation, and the final result is the average of ten evaluations. After parameter tuning, SVM is higher than other classifiers in most metrics, and reaches 90.0%, 0.952, 93.2%, 86.9%, 0.803, and 0.900% in ACC, AUC, SN, SP, MCC, and F1, respectively. Among all classifiers, ACC, AUC, SN, MCC, and F1 obtained by SVM achieved the first position. So we also focused on using SVM as a classifier for the independent test set.

**TABLE 2:** The results of cross-validation on the training set with different classifiers.

	Model	ACC (%)	SN (%)	SP (%)	AUC	MCC	F1
Training Set	SVM	**90.0**	**93.2**	86.9	**0.952**	**0.803**	**0.900**
	Bayes	86.5	83.2	87.9	0.865	0.729	0.838
	Knn	86.3	93.0	80.5	0.893	0.736	0.867
	DT	82.7	82.0	84.5	0.833	0.660	0.824
	RF	87.5	91.9	83.7	0.951	0.753	0.877
	Ada	82.2	84.8	79.8	0.823	0.645	0.822

The bold values indicate the best performance.

As can be seen from Table 3, SVM has a huge advantage over other classifiers on the independent test set V46p + 46n. The values of ACC, AUC, SN, SP, MCC, and F1 are 91.3%, 0.935, 97.8%, 84.8%, 0.833, and 0.918%, respectively. The values of ACC, AUC, SN, MCC, and F1 obtained by SVM all rank first among all classifiers. Especially MCC and AUC by SVM is 0.033 and 0.025 higher than the second-ranked classifier. The comparison of these results shows that SVM is the most suitable classifier in our work.

**TABLE 3:** The results of independent test on the testing set with different classifiers.

	Model	ACC (%)	SN (%)	SP (%)	AUC	MCC	F1
Testing Set	SVM	**91.3**	**97.8**	84.8	**0.935**	**0.833**	**0.918**
	Bayes	85.9	84.8	87.0	0.868	0.718	0.857
	Knn	89.1	97.8	80.4	0.910	0.800	0.900
	DT	82.6	80.4	84.8	0.826	0.653	0.822
	RF	88.0	93.5	82.6	0.931	0.765	0.887
	Ada	88.0	91.3	84.8	0.880	0.762	0.884

The bold values indicate the best performance.

**FIGURE 3:**
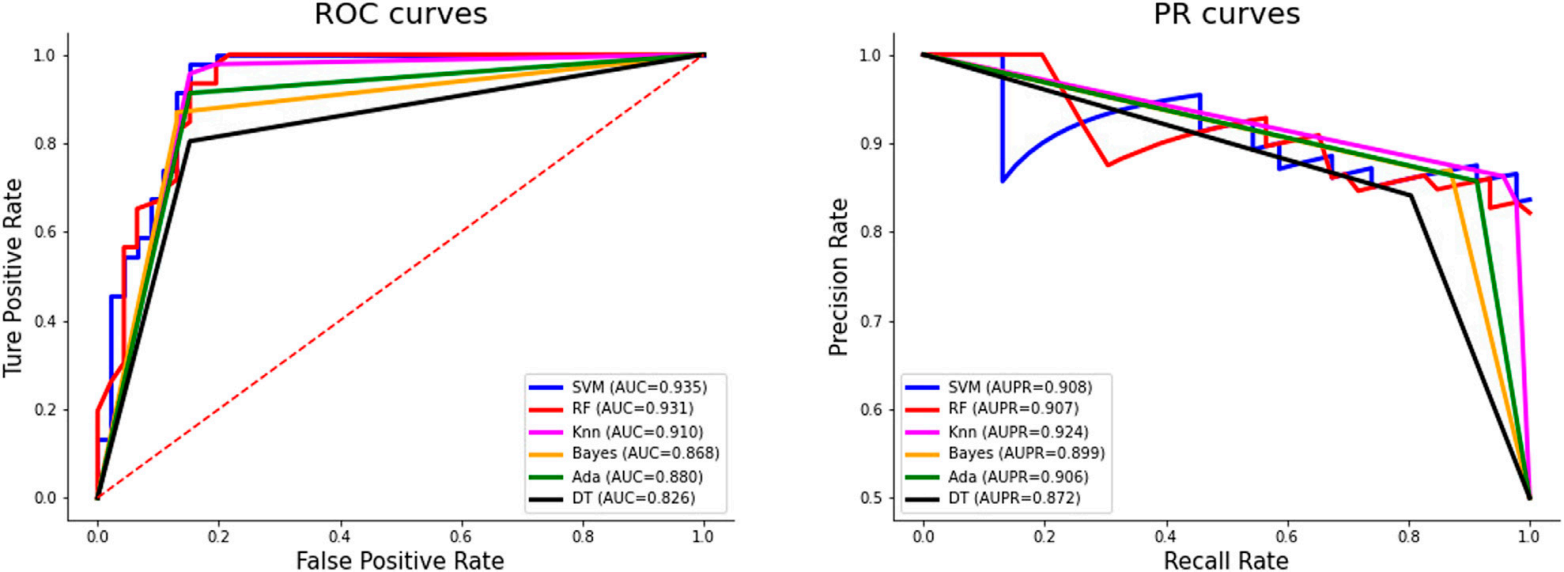
The performance of different classifiers through cross-validation on the training set.

Figure 3 shows the ROC curves and PR curves of different classifiers on the independent test set. The ROC curve of SVM is closest to the upper left corner, surpassing other classifiers. The AUC value of SVM is 0.935, which is the highest and 0.025 higher than the second-ranked classifier KNN. Although the AUPR value of SVM is not the largest, when the recall rate is 1, the precision rate of SVM reaches 0.836, which is the highest.

### Comparison With Other Methods

Our model is compared with others through ten-fold cross-validation on the training dataset, and the results are shown in Table 4. NM-BD and RUS-BD are both proposed in (Zhang et al., 2021), and the imbalanced training set was down sampled using NearMiss method (Mani and Zhang, 2003; Li et al., 2021) for the former, while the random under sampling method was used for the latter, which is also adopted in this study. Compared with RUS-BD, our model outperforms it on all metrics, with improvement of 1.8% on ACC, 0.7% on SN, 3% on SP, 1.8% on SP, 0.013 on F1, and 0.035 on MCC. When compared with NM-BD, our model is also the winner on nearly all metrics except SP. These results show that the performance of our model on the training set is better than the others on the whole.

**TABLE 4:** Comparison of our model with the existing methods through cross-validation on the training set.

Method	ACC (%)	SN (%)	SP (%)	MCC	F1
NM-BD	88.8	85.5	92.2	0.778	0.884
RUS-BD	88.2	92.5	83.9	0.768	0.887
**i2APP**	**90.0**	**93.2**	86.9	**0.803**	**0.900**

The bold values indicate the best performance.

To further verify the validity of the proposed model, we compare it with other models on an independent test dataset, and the results are shown in Table 5, from which we can see that the metrics of i2APP are nearly all better than that of other models. The values of ACC, SN, MCC and F1 are 17.4, 45.6, 0.302 and 0.251% higher than AMPfun, and the values of ACC, MCC, F1, and SP are 178 3.3, 0.107, 0.027, and 6.5% higher than PredAPP. All these results show that the proposed model has better generalization ability than the state-of-the-art models for APP prediction.

**TABLE 5:** Comparison of our model with the existing methods through independent test on the testing set.

Method	ACC (%)	SN (%)	SP (%)	MCC	F1
AMPfun	73.9	52.2	95.7	0.531	0.667
PredAPP	88.0	97.8	78.3	0.776	0.891
**i2APP**	**91.3**	**97.8**	84.8	**0.833**	**0.918**

The bold values indicate the best performance.

### Impact of Dataset Balancing

We performed 10-fold cross-validation on the original dataset containing 255 APPs and 1863 non-APPs, and the results were listed in Table 6. It can be found that compared with the balanced dataset, the SP, MCC and ACC metrics have a greater improvement on the unbalanced dataset. However, because there are too few positive samples, the SE metric decreases a lot. In addition, our model achieves large improvements in various metrics compared to the model PredAPP (IMBD) (Zhang et al., 2021) using the same unbalanced dataset.

**TABLE 6:** The results of ten-fold cross-validation on the balanced or unbalanced datasets.

Method	ACC (%)	SN (%)	SP (%)	MCC	F1
PredAPP (unbalanced)	91.9	52.5	97.3	0.574	0.609
i2APP (balanced)	90.0	93.2	86.9	0.803	0.900
i2APP (unbalanced)	96.5	76.7	99.3	0.826	0.839

With the unbalanced dataset as the training set, we tested the proposed model on the independent test set including 46 APPs and 46 non-APPs and listed the results in Table 7, from which we can see that whether using balanced or unbalanced training sets, i2APP has good generalization ability.

**TABLE 7:** The results of independent test using the balanced or unbalanced datasets as the training set.

Method	ACC (%)	SN (%)	SP (%)	MCC	F1
i2APP (balanced)	91.3	97.8	84.8	0.833	0.918
i2APP (unbalanced)	93.5	100.0	87.0	0.877	0.939

### Impact of Shuffled Sequence

**FIGURE 4:**
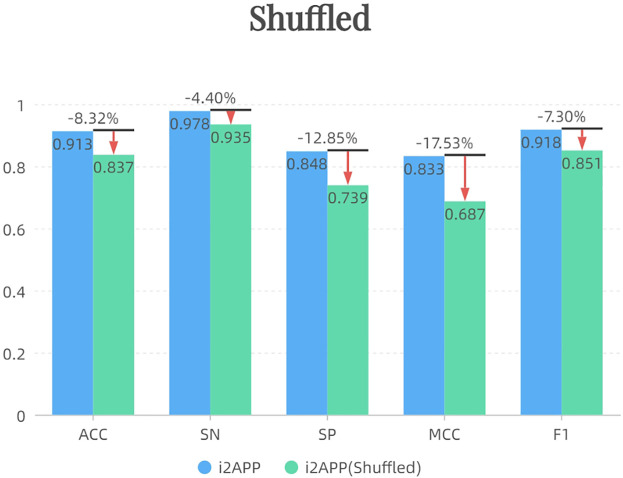
The effect of shuffling the sequence.

After shuffling the sequence of negative samples in the training set, we randomly sampled 255 new negative samples to form the training set together with 255 positive samples. The results of independent test are shown in Figure 4. It can be seen that the performance of the model decreases after using the shuffled negative samples, probably because the effect of the terminus-based features is reduced after the sequence is shuffled.

### Interpretability Analysis

**FIGURE 5:**
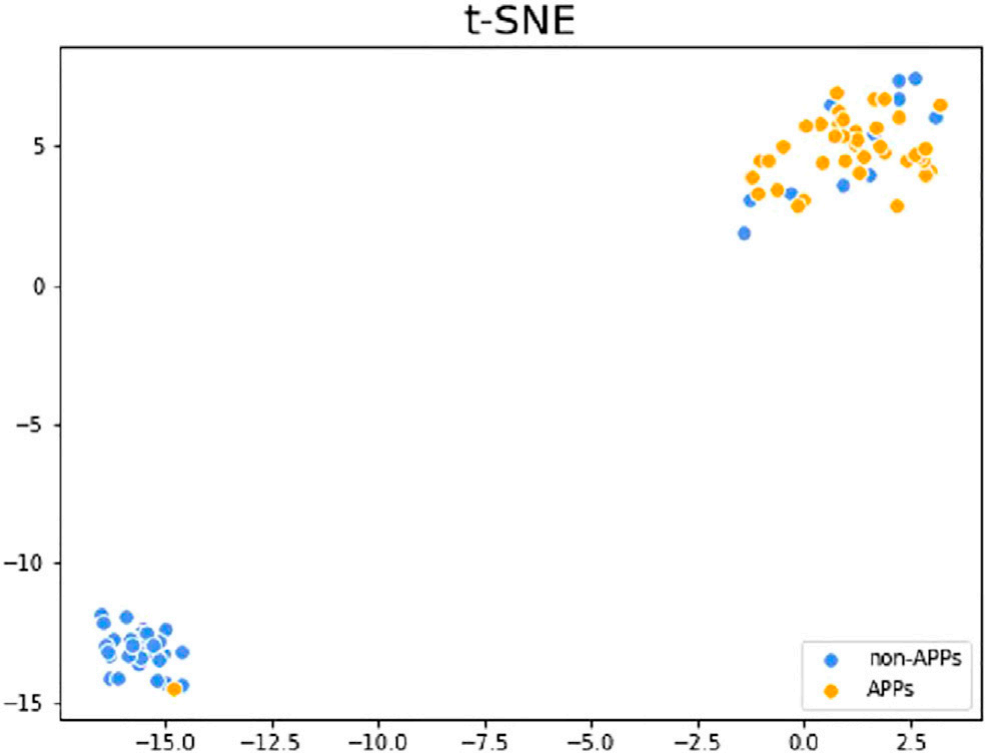
*t*-SNE visualization results of the testing set after dimensionality reduction of the higher-level features.

T-distributed stochastic neighbor embedding (t-SNE) (Van der Maaten and Hinton, 2008) is a very popular data visualization tool that can reduce high-dimensional data to 2-3 dimensions, so as to draw samples on a plane or 3D space and observe the sample distribution. Figure 5 shows the visualization results of the test dataset V46p + 46n after dimensionality reduction on the higher-level features, which are the outputs of the first layer classification. The orange points in the figure are APPs, and the blue points are non-APPs. As can be seen from the figure, the two types of samples can be well distinguished with the higher-level features, so that our model can achieve better performance. What’s more, it can be found that the aggregation degree of APPs is higher than that of non-APPs, indicating that it is easier to identify APPs than non-APPs, so the metric SN in our model will be higher than SP.

## Conclusion

In this study, we propose a novel model named i2APP to identify APPs efficiently. The main structure of this work consists of four steps. Firstly, the random under sampling method is used to balance the training set. Secondly, a variety of sequence-based and terminus-based features are extracted from any peptide sequence, and then enter these raw features into the first layer classifiers, SVM and LGBM, to get the higher-level features. The maximum information coefficient (MIC) is calculated for each higher-level feature, and only the significant features are retained. Thirdly, based on the optimal feature subset, several popular classifiers are evaluated through cross-validation on the training dataset, and SVM is chosen as the second layer classifier. Finally, independent test is performed on the proposed model and the others, and we can see that i2APP has better generalization ability than the state-of-the-art models for APP prediction. The sequence features used in this paper are all extracted by hand, and some of them are quite complex. Although we simplify the model by two-step learning and feature selection, the overall model still looks complicated. In the future, as the amount of data increases, the RNN or Transformer model can be used for automatic feature learning, which may further improve the accuracy of APP recognition.

## Data Availability

Publicly available datasets were analyzed in this study. This data can be found here: https://github.com/greyspring/i2APP/tree/master/datasets.
